# Conjugates of cytochrome *c* and antennapedia peptide activate apoptosis and inhibit proliferation of HeLa cancer cells

**DOI:** 10.3892/etm.2013.1205

**Published:** 2013-07-04

**Authors:** PATRICK IMESCH, DAVID SCHEINER, EMESE SZABO, DANIEL FINK, ANDRÉ FEDIER

**Affiliations:** Department of Gynecology, University Hospital Zurich, Zurich CH-8091, Switzerland

**Keywords:** apoptosis, cell-penetrating peptides, cytochrome *c*, proliferation

## Abstract

Polycationic cell-penetrating peptides (CPPs) deliver macromolecules into cells without losing the functional properties of the cargoed macromolecule. The aim of this study was to determine whether exogenous cytochrome *c* is delivered to HeLa cervical carcinoma cells by the CPP antennapedia (Antp) and activates apoptosis. HeLa cervical carcinoma cells were treated with conjugated Antp-SMCC-cytochrome *c* (cytochrome *c* chemically conjugated to Antp) or with non-conjugated Antp and cytochrome *c*. Sensitivity to the treatments was determined by the clonogenic assay (proliferation) and by immunoblot analysis (apoptosis activation). We report that conjugated Antp-SMCC-cytochrome *c* activated apoptosis in HeLa cells as demonstrated by poly (ADP-ribose) polymerase 1 (PARP-1) cleavage and inhibited their proliferation. The Antp-SMCC-cytochrome *c*-induced apoptosis was inhibited by z-VAD-fmk, a pan-caspase inhibitor peptide. Unconjugated Antp or cytochrome *c* demonstrated no inhibitory effect on survival and proliferation. Our results suggest that chemical coupling of cytochrome *c* to CPPs may present a possible strategy for delivering cytochrome *c* into cells and for activating apoptosis.

## Introduction

Cytochrome *c* is a highly conserved, water-soluble protein of 12.3 kD with a net positive charge at neutral pH, residing loosely attached in the mitochondrial intermembrane space. It has a dual function; it is involved in energy production in mitochondria by interaction with redox partners and it also has a critical function in the induction of intrinsic (cytochrome *c*/mitochondria-mediated) apoptosis. Intrinsic apoptosis is activated by cellular stress originating from inside the cell (e.g. DNA damage or the presence of reactive oxygen species) and is strictly dependent on the release of cytochrome *c* from the mitochondria into the cytoplasm upon an intrinsic (i.e. of intracellular origin) stimulus. Cytochrome *c* is then, together with other cytosolic factors, including apoptotic protease activating factor 1 (Apaf-1) and pro-caspase-9, assembled into the apoptosome. Following apoptosome assembly and activation of pro-caspase-9 (initiator caspase), the downstream caspases-3 and −7 (effector caspases) are cleaved and thereby activated. This leads to the execution of the apoptotic program, culminating in the dismantling of the cell (1–4). Apoptosis also occurs through the extrinsic (cytochrome *c*-independent) Fas/FasL-mediated pathway, which merges with the intrinsic pathway at the level of the effector caspases-3 and −7 (5).

The findings that exogenous cytochrome *c*, either microinjected directly into the cytoplasm or delivered into the cytoplasm by electroporation, activates apoptosis without the requirement for additional apoptotic stimuli supports the critical role of cytochrome *c* in apoptosis (6–8). Related studies have demonstrated that apoptosis in tumor cells is activated by cytochrome *c* delivered by nanoparticles, including nanotubes or polylactic-co-glycolic acid (PLGA) microspheres (9,10). This suggests that the cytoplasmic delivery of exogenous cytochrome *c* through suitable carriers with subsequent apoptosis activation is a potential therapeutical approach against cancer. Contrary to necrosis, apoptosis does not induce an immune response of the surrounding tissue, which may be of clinical significance.

Cell-penetrating peptides (CPPs) are a group of peptides that are often ~20 amino acids long and contain a cluster of basic residues. Based on their property of translocating across the hydrophobic cell membrane, they are also capable of delivering protein- and DNA-based macromolecules and drug molecules to cells without the loss of biological activity of the conveyed materials. CPPs are intensively studied and considered as important carriers in drug delivery (11–15).

Antennapedia (Antp) is one member of the family of CPPs. Antp was originally derived from the 60 amino acid long homeodomain of the Drosophila transcription factor Antennapedia (16). Later on, its translocation ability was narrowed down to a 16-mer, termed as penetratin (Antp PTD, 43–58 residues, RQIKIWFQNRRMKWKK) present in the homeodomain (17). In the present study we describe the effects of Antp-SMCC-cytochrome *c*, a conjugate molecule synthesized from cytochrome *c* and Antp on apoptosis activation and proliferation inhibition in HeLa cervical tumor cells.

## Materials and methods

### Cell culture and compounds

HeLa cervical cancer cells (obtained from Dr G. Marra, Institute of Molecular Cancer Research, University of Zurich) were routinely cultured in Iscove's modified Dulbecco's medium (IMDM)-21980 (Invitrogen, Basel, Switzerland) containing 10% fetal calf serum (Oxoid, Basel, Switzerland) at 37°C and in an atmosphere of 5% carbon dioxide and 95% humidity. Horse heart cytochrome *c* was purchased from Sigma-Aldrich Chemie GmbH (Buchs, Switzerland) and a stock solution (20 mg/ml, 1.63 mM) was prepared in sterile water and stored at −20°C. The 19-mer synthetically synthesized Antp peptide was purchased from Bachem (Bubendorf, Switzerland) and solutions were prepared in phosphate-buffered saline (PBS) containing 2 mM tributylphosphine prior to use. This Antp peptide (amino acid sequence, Ser-Gly-Arg-Gln-Ile-Lys-Ile-Trp-Phe-Gln-Asn-Arg-Arg-Met-Lys-Trp-Lys-Lys-Cys) was biotinylated at the 5′-carboxy terminus and functionalized at the 3′-amino terminus with a trifluoroacetate group. Sulfo-succinimidyl 4-(N-maleimidomethyl)cyclohexane-1-carboxylate (SMCC) was purchased from Pierce Biotechnology Inc. (Lausanne, Switzerland) and solutions were freshly prepared in PBS. The pan-caspase inhibitor peptide z-VAD-fmk was purchased from Enzo Life Sciences (Laufen, Switzerland) and a stock solution in dimethyl sulfoxide (DMSO) was stored at −20°C.

### Conjugate synthesis

The Antp-SMCC-cytochrome *c* conjugate synthesis was a two-step reaction, where sulfo-SMCC was used as a cross-linker molecule (also referred to as a bifunctional coupling reagent). The conjugate synthesis was performed as follows: In the first step, cytochrome *c* was incubated with crystalline sulfo-SMCC in PBS at a molar ratio of protein molecules to succinimidyl groups of 1:4 for 60 min under continuous stirring at room temperature. This coupled the sulfo-SMCC covalently to cytochrome *c*. Excess sulfo-SMCC was removed by overnight dialysis at 4°C against PBS. In the second step, the sulfo-SMCC-coupled cytochrome *c* was incubated with freshly prepared Antp solution containing 2 mM tributylphosphine (to prevent dimerization of the Antp peptides) at a molar ratio of cytochrome *c*-SMCC:Antp of 1:5 for 48 h under continuous stirring at 4°C. The reddish conjugate solution was then filtered [Millex-HV polyvinylidene fluoride (PVDF) 0.45-μm pore-size sterile filter]. The concentration of cytochrome *c* in the conjugate was determined by a cytochrome *c* (human) enzyme-linked immunosorbent assay (ELISA) kit (Enzo Life Sciences) according to the manufacturer's instructions.

### Cell lysates and immunoblot analysis

Immunoblot analysis was performed in cell lysates to assess apoptosis on the basis of the treatment-induced proteolytic cleavage of the 116 kDa PARP-1 precursor into its 89 kDa fragment. Proteolytic PARP-1 cleavage is an acknowledged measure of ongoing apoptosis. Cell lysates were produced from untreated HeLa control cultures or HeLa cultures treated with either the Antp-SMCC-cytochrome *c* conjugate or the non-conjugated compounds (cytochrome *c*, Antp) for 24 h, washed in PBS and lysed according to standard laboratory protocols. In certain cultures the pan-caspase inhibitor peptide z-VAD-fmk was added (10 or 20 μM) 2 h before the addition of Antp-SMCC-cytochrome *c*. The protein concentration of cell lysates was determined using the BCA Protein Assay kit (Pierce Biotechnology Inc.). For immunoblot analysis (performed following standard laboratory protocols), 20 μg cell lysate protein was separated using 10% sodium dodecyl sulfate-polyacrylamide gel electrophoresis (SDS-PAGE), followed by blotting onto a PVDF membrane (Amersham Biosciences, Otelfingen, Switzerland). Proteins were detected by the specific primary antibodies and the respective secondary antibodies: horseradish peroxidase (HRP)-conjugated anti-mouse (M15345; BD Transduction Laboratories, Lexington, KY, USA) or HRP-conjugated anti-rabbit (7074, Cell Signaling Technology Inc./BioConcept, Allschwil, Switzerland). The primary antibodies used were PARP-1 (9542, Cell Signaling; recognizing the 116 kDa full-length PAPR-1 and the cleaved 89 kDa fragment) and anti-mouse β-actin (A5441, Sigma) or anti-rabbit α/β-tubulin (2148, Cell Signaling) as sample loading controls. Complexes were visualized by enhanced chemiluminescence (Amersham Biosciences) and autoradiography. A HeLa cell culture treated with 0.8 mM H_2_O_2_ for 6 h served as the positive control sample for apoptosis.

### Clonogenic assay

The sensitivity of HeLa cells to the treatments was determined by the clonogenic assay. HeLa cells (500 cells in 2 ml culture medium) were plated in 35 mm cell culture plates. Then, 24 h after plating, the cells were treated with various concentrations of either the conjugate or the non-conjugated compounds for 24 h. Then, the drug-containing medium was replaced with drug-free medium. Seven days after treatment, cells were fixed with 25% acetic acid in ethanol and stained with Giemsa. Colonies of ≥50 cells were scored visually. Each experiment was performed three times. Clonogenic survival was presented as the percentage of the untreated control as a function of the compound concentration.

## Results

### Antp-SMCC-cytochrome c conjugate activates caspase-dependent apoptosis

**Figure 1:**
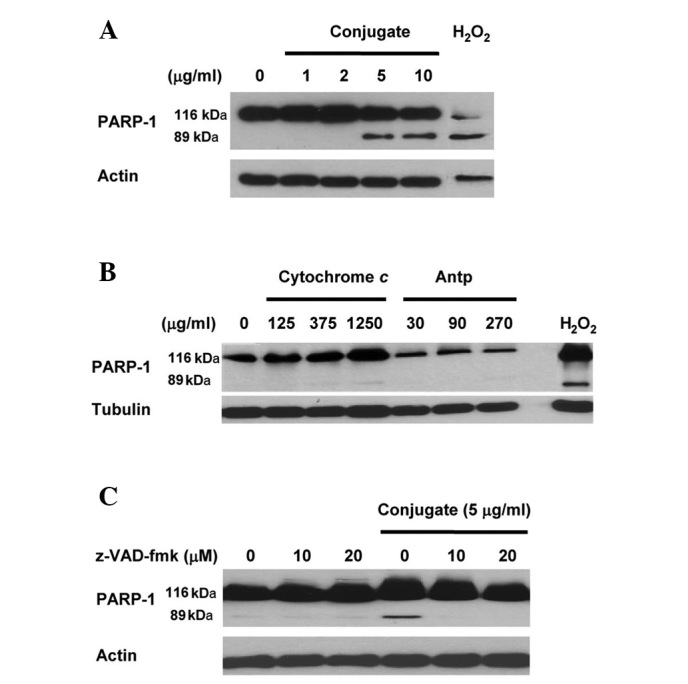
Effects of a 24-h treatment with (A) the Antp-SMCC-cytochrome *c* conjugate (1, 2, 5 or 10 μg/ml) and (B) non-conjugated cytochrome *c* (125, 375 or 1250 μg/ml) or non-conjugated Antp (30, 90, or 270 μg/ml) on apoptosis (i.e. proteolytic cleavage of the 116-kDa PARP-1 precursor into a 89-kDa fragment) in HeLa cells. (C) Also shown is the effect of the pan-caspase inhibitor z-VAD-fmk (10 and 20 μM; 2 h pretreatment) on apoptosis (cleavage of the 116 kDa PARP-1 precursor into the 89 kDa fragment) of HeLa cells treated with 5 μg/ml Antp-SMCC-cytochrome *c* conjugate for 24 h. HeLa cells treated with 0.8 mM hydrogen peroxide (H_2_O_2_) for 6 h served as the positive control sample for apoptosis. Actin and tubulin are the sample loading controls. Data are the representatives of two independent experiments. Antp, antennapedia; PARP-1, poly (ADP-ribose) polymerase 1.

Immunoblot data (Fig. 1A) revealed that, in comparison with the untreated control sample, the treatment of HeLa cells with Antp-SMCC-cytochrome *c* resulted in the cleavage of the 116-kDa PARP-1 precursor into an 89-kDa cleaved fragment (a measure for ongoing apoptosis). A concentration of cytochrome *c* (contained in the conjugate and measured by cytochrome *c*-specific ELISA) as low as 5 μg/ml was sufficient to result in PARP-1 cleavage, i.e. to activate apoptosis. By contrast, PARP-1 cleavage was not observed when HeLa cells were treated with either cytochrome *c* or Antp alone at concentrations of up to 1,250 μg/ml or 270 μg/ml, respectively (Fig. 1B). This indicates that apoptosis is activated by treatment with the Antp-SMCC-cytochrome *c* conjugate but not with Antp or cytochrome *c* alone.

The 2-h pretreatment of HeLa cultures with 10 or 20 μM z-VAD-fmk and the subsequent treatment with Antp-SMCC-cytochrome *c* (5 μg/ml) eliminated the Antp-SMCC-cytochrome *c*-induced apoptosis. This was manifested by the failure to detect PARP-1 precursor cleavage (Fig. 1C). As a broad spectrum caspase inhibitor peptide, z-VAD-fmk irreversibly inhibits the activity of the majority of the members of the caspase-family, indicating that the Antp-SMCC-cytochrome *c*-induced apoptosis was caspase-dependent.

### Antp-SMCC-cytochrome c conjugate inhibits clonogenic survival

**Figure 2:**
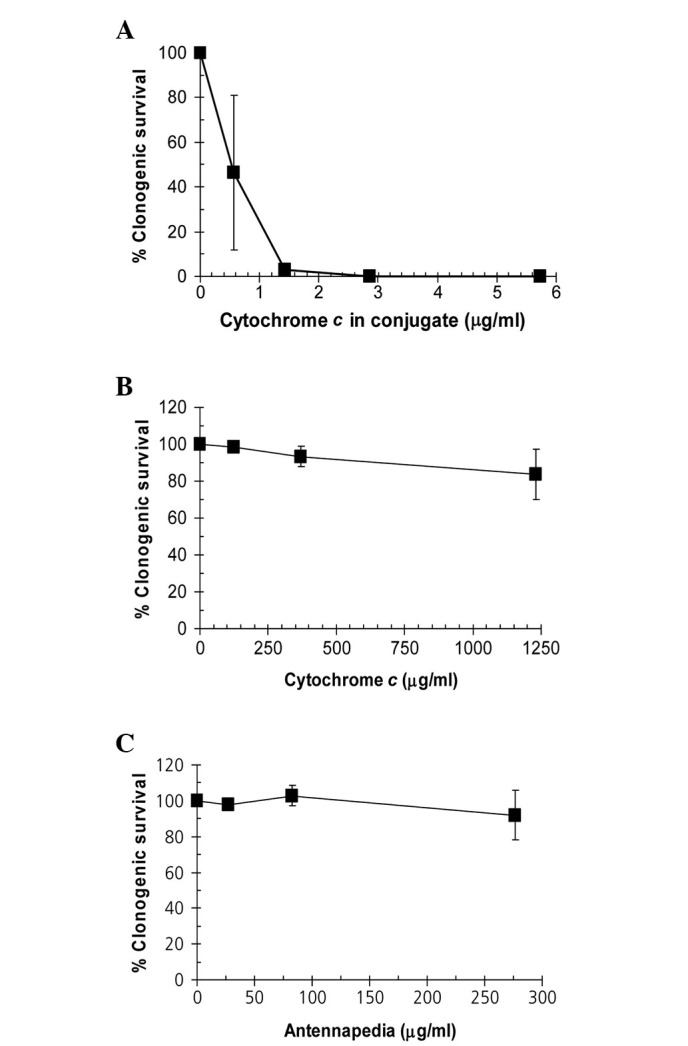
Effects of a 24-h treatment with (A) the Antp-SMCC-cytochrome *c* conjugate, (B) non-conjugated cytochrome *c* or (C) non-conjugated Antp on the clonogenic survival of HeLa cells. Data points are the mean ± SD of three independent experiments performed in triplicate cultures. Antp, antennapedia; SD, standard deviation.

The Antp-SMCC-cytochrome *c* conjugate reduced the clonogenic survival of Hela cells (Fig. 2A). A concentration as low as 1.3 μg/ml cytochrome *c* (contained in the conjugate) was sufficient to completely block the clonogenic potential of HeLa cells. By contrast, cytochrome *c* alone (≤1,250 μg/ml) or Antp alone (≤275 μg/ml) did not produce a substantial negative effect on clonogenic survival (Fig. 2B and C).

## Discussion

Cytochrome *c* has been shown to activate apoptosis when directly microinjected or delivered into tumor cells via electroporation or nanoparticles. CPPs, including Antp, facilitate the penetration of various biomolecules and particles into cells. On this basis, we synthesized the conjugate molecule Antp-SMCC-cytochrome *c* from the respective compounds (cytochrome *c* and Antp) using the sulfo-SMCC crosslinker and determined the effects of this Antp-SMCC-cytochrome *c* conjugate on survival, i.e. apoptosis activation and proliferation in HeLa cervical cancer cells.

The aim of the present study was to determine whether apoptosis in HeLa tumor cells is activated by exogenous cytochrome *c* delivered into the cytoplasm through the CPP Antp in the form of a conjugate molecule consisting of Antp covalently linked to cytochrome *c*.

In the current study, we demonstrated that cytochrome *c* covalently conjugated to Antp applied to HeLa cervical cancer cell cultures activates caspase-dependent apoptosis and inhibits proliferation, whereas neither cytochrome *c* nor Antp alone affected survival and proliferation. Therefore, we conclude that the inhibitory effects on survival and proliferation are attributed to cytochrome *c* delivered to HeLa cells via Antp. This suggests that the Antp-aided delivery of cytochrome *c* into tumor cells may be a candidate strategy for activating apoptosis and consequently inhibiting the survival and proliferation of tumor cells.

In a pilot set of experiments, we demonstrated that the presence of non-conjugated cytochrome *c* alone in the culture medium did not activate apoptosis nor substantially reduce the clonogenic potential at concentrations of up to 1,250 μg/ml, suggesting that cytochrome *c* is not accumulated in the cytoplasm. This suggestion is supported by findings that cytochrome *c* is unable to translocate across membranes on its own and therefore requires the so-called translocases in the outer membrane (TOM) complex for the translocation across the mitochondrial outer membrane (18). The presence of (non-conjugated) Antp (concentrations ≤270 μg/ml) alone in the culture medium had no effect on apoptosis and clonogenic potential. This suggests that Antp is not harmful in this experimental setting. It is known that CPPs are toxic to cells due to membrane perturbation at higher levels of the peptides (19).

The key finding in the present study was that, unlike non-conjugated cytochrome *c* and Antp, the incubation of HeLa cultures with the Antp-SMCC-cytochrome *c* conjugate resulted in the activation of apoptosis and reduction of the clonogenic potential of HeLa cells. Antp-SMCC-cytochrome *c*-induced apoptosis is caspase-dependent, since it was inhibited by the pan-caspase inhibitor z-VAD-fmk.

The following series of events that eventually lead to apoptosis may be proposed on the basis of the results of the current study. The Antp-SMCC-cytochrome *c* conjugate translocates across the cellular membrane and accumulates in the cytoplasm, where the conjugate is hydrolyzed into its components (the SMCC-crosslinker is pH-sensitive). Cytochrome *c* is then assembled into the apoptosome that, in turn, finally results in the activation and the execution of apoptosis. This implies that the structural integrity and the biological function of cytochrome *c* are not compromised by the chemical modifications made during Antp-SMCC-cytochrome *c* conjugate synthesis and its subsequent hydrolysis. Studies have shown that injection of ~10 fg cytochrome *c* is sufficient to activate apoptosis (6), corresponding to an estimated intracellular cytochrome *c* concentration of ~20 μM (7). Whether and to what extent cytochrome *c* molecules with covalently bound SMCC retain functional integrity in terms of proper apoptosome formation remains unclear. Likewise, the possible effects of the other products of the hydrolysis with respect to apoptosis activation and clonogenic survival are unknown, but may be marginal.

It is important to acknowledge that the results of the present study should be considered as proof-of-concept only, and that more detailed studies should be performed. However, hypotheses towards important features related to antitumor studies may be proposed.

Conventional chemotherapy is an indispensable therapeutic option for the treatment of a number of malignancies. It kills tumor cells through the activation of the apoptotic machinery by the use of foreign-to-body chemicals or biological compounds. These compounds are by definition toxic and are frequently of limited bio-tolerability and bio-degradability. Clinicians and patients are therefore often confronted with limitations, including adverse side-effect profiles. Cytochrome *c* as the therapeutically active compound against tumor cells appears appealing and may be a candidate alternative to conventional chemotherapy. It is intrinsic to cells and not toxic; however, it is able to activate apoptosis when delivered to cells from outside in femtogram quantities.

Exogenous cytochrome *c* as the ‘therapeutically’ active compound may help overcome certain types of chemotherapy resistance. Resistance to chemotherapeutic compounds emerges through the expression of multidrug resistance drug efflux transporters or drug detoxifiers, or through the enhanced repair of damaged DNA (20). This leads to ineffective mitochondrial cytochrome *c* release due to the absence of apoptotic stimuli, to ineffective apoptosome assembly and caspase activation, and eventually to ineffective apoptosis execution. Absent release of intrinsic cytochrome *c* may be compensated by the exogenously delivered cytochrome *c*, thereby overcoming chemoresistance. It may also be hypothesized that exogenous cytochrome *c* does not cause the acquisition of drug resistance in tumor cells, a major problem of conventional chemotherapies.

Despite its intriguing characteristics, there are critical issues with the concept of CPP-aided cytochrome *c* delivery. One is that CPPs have limited target specificity; CPPs are likely to deliver their cargo not only to tumor cells, but also to normal cells. Further studies are required to render CPP-aided delivery target cell-specific. An alternative to CPP-aided cytochrome *c* delivery may be cytochrome *c* delivery via tumor cell-targeted immunoliposomes; however, this approach may suffer from limitations associated with the intrinsic disadvantages of endocytotic-based mechanisms. Another issue is what the potential clinical application of the CPP-aided cytochrome *c*-therapy may be. We performed this study with HeLa cervical cancer cells; therefore, it may be applied as a therapy of inoperable, local cervical cancers or advanced primary inoperable vulvar and vaginal cancers that are easily accessible to, for instance, an Antp-SMCC-cytochome c-containing ointment. A similar application may also be suitable for superficial cancers, including skin cancer.
